# The Association of Hypertension Based on Systolic and Diastolic Blood Pressure (SBP and DBP) With Dental Visits in the Population Aged 45 and Older: Cross‐Section Study Results From the China Health and Retirement Longitudinal Study (CHARLS)

**DOI:** 10.1002/clc.70406

**Published:** 2026-07-09

**Authors:** Man Ao, Wei Li, Shiyi Luo, Jukun Song, Xuanyan Gu, Jiangling Sun, Zhu Chen

**Affiliations:** ^1^ Guizhou University Medical College Guiyang Guizhou province China; ^2^ Guiyang Hospital of Stomatology Guiyang Guizhou province China; ^3^ Department of Oral and Maxillofacial Surgery the Affiliated Stomatological Hospital of Guizhou Medical University Guiyang Guizhou province China

**Keywords:** CHARLS, cross‐sectional study, hypertension, oral health

## Abstract

**Objective:**

Systemic diseases increasingly complicate oral treatment, particularly in hypertensive patients requiring comprehensive care. Using nationally representative cross‐sectional data, this study investigated the association between hypertension and oral health in Chinese adults aged ≥ 45 years.

**Methods:**

Data were drawn from the 2013 and 2015 CHARLS, including 4770 participants aged ≥ 45 years. Linear and logistic regression examined blood pressure–oral disease associations, with systolic/diastolic pressure first as continuous, then categorical variables. Curve fitting and restricted cubic splines further validated the cross‑sectional associations.

**Results:**

A total of 4770 participants were included in this study, including 1568 patients with hypertension. The characteristics of the hypertensive population are married elderly men who enjoy smoking and drinking, suffer from other chronic diseases, and low to moderate level of self‐reported health. Logistic regression revealed positive associations of systolic and diastolic blood pressure with dental visits, and a stable positive correlation between hypertension and dental visits persisted across multiple adjustment models, further validated by curve fitting and restricted cubic spline analyses.

**Conclusion:**

Hypertension is significantly and positively associated with oral health issues in a nationally representative sample of middle‐aged and older Chinese adults. Healthcare providers should prioritize integrated blood pressure and oral health management in this population to mitigate the mutual exacerbation of chronic diseases.

## Graphical abstract

There is a significant positive correlation between hypertension and dental visits among adults aged 45 and above in China. This suggests that attention should be paid to the coordinated management of cardiovascular health and oral health to prevent the mutual aggravation of chronic diseases.

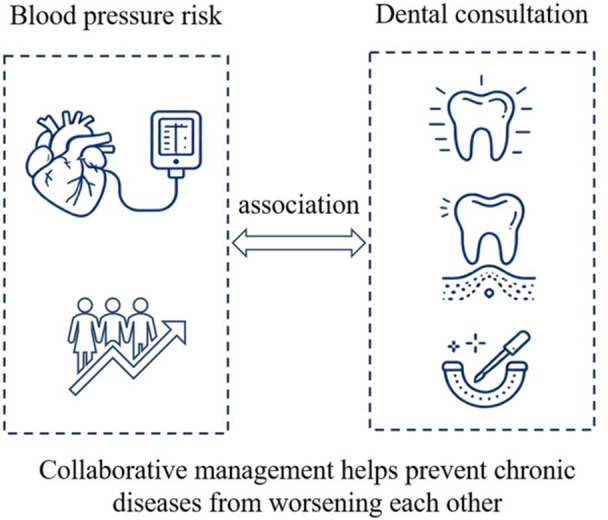

**Abbreviations**
95% Cl: 95% confidence interval
CESD: Center for Epidemiologic Studies Depression Scale
CHARLS: China Health and Retirement Longitudinal Study
DBP: diastolic blood pressure
SBP: systolic blood pressure
SD: standard deviation
STROBE: STrengthening the Reporting of OBservational studies in

## 1 Introduction

With China's entry into an aging society, the incidence rate of oral diseases among the elderly is also increasing, and elderly patients with oral diseases often suffer from systemic diseases, with multiple diseases intersecting with each other [1, 2, 3]. Their diagnostic and treatment processes are more complex, and they are also accompanied by high risks. Hypertension is the most prevalent cardiovascular disease, accounting for 45% of the total cardiovascular mortality rate, according to data from the World Health Organization [4]. Hypertension is a complex multifactorial disease, and no simple mechanism can fully explain the increase in blood pressure [5]. In clinical practice, blood pressure is an important predictor of cardiovascular risk, and blood pressure indicators, such as systolic and diastolic blood pressure (DBP), are often reliable factors for determining the level of hypertension [6]. However, in China, the incidence and prevalence of hypertension are high, reflecting the challenges associated with cardiovascular health. Alarmingly, many people remain unaware of their hypertension, and the rates of treatment and control are low. This leads to ineffective management of the disease and an increased risk of cardiovascular disease [7].

At present, oral diseases have become a global public health issue, with approximately 55% of the population suffering from varying degrees of oral diseases, especially in the elderly population, where oral diseases have become one of the main disease burdens [2]. The results of the Fourth National Oral Epidemiological Survey in China show that middle‐aged and elderly residents have weak awareness of oral health care, and problems such as periodontal disease, dental arch defects, and dental caries are more prominent [8, 9, 10]. Oral diseases not only affect the physiological functions of the mouth, such as chewing and pronunciation, but are also closely related to systemic diseases, such as diabetes and cardiovascular diseases, endangering the quality of life of middle‐aged and elderly people [11, 12].

Clinical reports have indicated a correlation between oral diseases and hypertension [13]. Researchers have also explored the link between oral microbiota and hypertension, suggesting a potential association between oral inflammation and vascular diseases [14]. Some scholars believe that the mechanism may be that inflammatory factors in the local periodontal environment of patients can enter the bloodstream and be transmitted throughout the body, causing systemic chronic inflammation and subsequently increasing the risk of cardiovascular disease or oral health [12, 15]. Therefore, understanding the impact of oral health on hypertension is crucial for comprehensive management of cardiovascular risk. Despite the significance of this relationship, there is relatively little research in China examining the association between dental visits and hypertension based on large sample sizes [16]. To address this gap, the current study employed a cross‐sectional research design to analyze data from the China Health and Retirement Longitudinal Study (CHARLS), aiming to explore the association between hypertension and dental visits among individuals aged ≥ 45 years. This study aimed to evaluate whether the number of dental visits is related to the risk of hypertension in the middle‐aged and elderly population. Given the increasing prevalence of oral diseases and hypertension in China, this study may provide a theoretical basis for developing more effective public health strategies and clinical interventions to address these intertwined chronic health issues.

## 2 Methods

### 2.1 Population and Study Design

The CHARLS was established in 2011 and is an ongoing longitudinal survey representative of China. It collects high‐quality data through one‐on‐one, structured questionnaires. The respondents were Chinese people aged 45 years and above, who were sampled by multistage stratified probability in proportion. All participants were evaluated using standardized questionnaires to collect data on sociodemographic and lifestyle factors, as well as health‐related information [17]. These data include individually weighted variables to ensure that the survey sample is nationally representative. This study used data from the second and third waves of CHARLS (2013−2015), which can be found at https://charls.pku.edu.cn/ Obtain). Chinese residents aged 45 and above from urban and rural areas in 28 provinces were randomly selected. This study was a multicenter cohort study conducted by the Guidelines for Reporting Observational Studies in Enhanced Epidemiology (STROBE), in compliance with the Helsinki Declaration, and approved by the Institutional Review Board of Peking University (IRB00001052‐1015). The survey was conducted with all participants signing informed consent forms [18].

**Figure 1:**
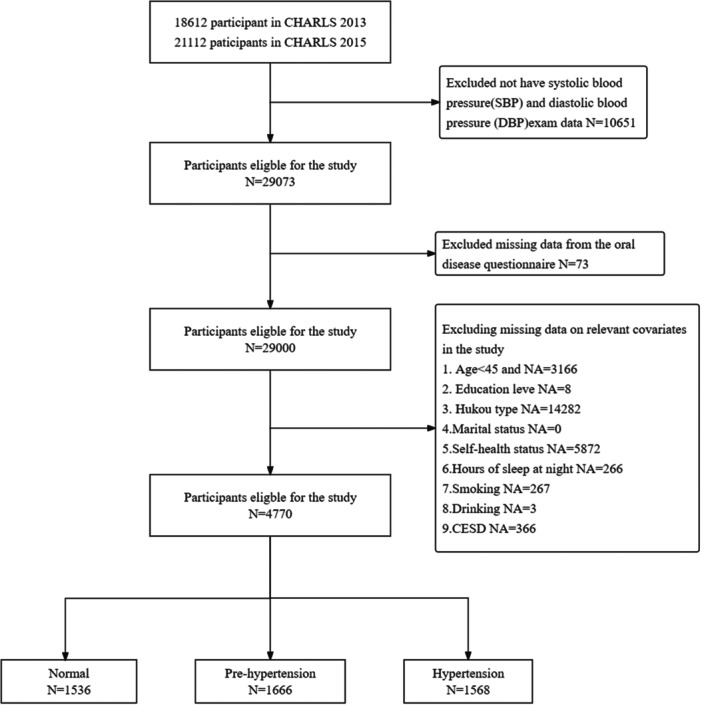
Sample selection flowchart for this study.

In this study, we conducted a cross‐sectional analysis of the merged data *n* = 39,724 from two CHARLS cycles in 2013 (wave 2 = 18,612) and 2015 (wave 3 = 21,112). The inclusion criteria for this study were as follows: (1) individuals aged at least 45 years in the CHARLS study conducted from 2013 to 2015. (2) having data on visits to hospitals due to oral diseases within the past year. (3) having complete blood pressure monitoring data from three times. The exclusion criteria are: (1) *n* = 10,651 missing blood pressure measurement data and *n* = 73 missing oral disease data in CHARLS data from 2013 to 2015, (2) No age information, *n* = 2399, (3) Individuals under the age of 45, *n* = 767, (4) Covariant data missing *n* = 21,064. The specific flow chart of the acceptance and discharge standards is shown in Figure 1.

### 2.2 Definition of Exposure and Outcome

#### 2.2.1 Assessment of Hypertension

After resting quietly in a sitting position for 5 min, participants used an effective oscilloscope device (Omron HEM‐7200 Monitor) on their right arm to measure blood pressure. Three measurements were taken every minute, and the average of the three measurements of systolic blood pressure (SBP) and DBP were used as the final blood pressure value. Participants who appeared in both waves were represented only by their most recent observation. Each participant contributed exactly one observation to the final analytical dataset, ensuring independence of observations. According to the sixth report of the Joint Committee on Prevention, Testing, Evaluation, and Treatment of Hypertension, the diagnostic criteria for hypertension are that the average DBP measurement reading is ≥ 90 mmHg and the average SBP measurement reading remains ≥ 140 mmHg in at least two or more subsequent visits [19]. This study classified blood pressure based on the SBP and DBP cut‐off points proposed in the JNC7 report [20]. Pre‐hypertension is defined as 120 ≤ SBP < 140 mmHg or 80 ≤ DBP < 90 mmHg, while hypertension is defined as an average SBP exceeding 140 mmHg or DBP exceeding 90 mmHg or medication treatment for hypertension [21].

#### 2.2.2 Dental Visits

The oral data of the participants comes from the questionnaire data during the second and third waves of the survey, and the question is: “Can you know about your dental visits in the past year.” The criterion for determining whether middle‐aged and elderly people have suffered from oral diseases was whether they had visited medical institutions for oral diseases in the past year. This study selected data waves from 2013 to 2015 because dental variables (dental visits) and blood pressure were only available during these two cycles. The datasets were publicly available and could be accessed from the CHARLS homepage at http://charls.pku.edu.

#### 2.2.3 Covariates

A structured survey questionnaire was used to collect information on sociodemographic status and health‐related factors. There are five sociodemographic variables, including age, gender, and educational level (divided into: illiterate, primary (include literate), high school and above), marital status (divided into: married with spouse present, married not living with spouse, separated/divorced/widowed/never or cohabitated) and Hukou type (divided into: agricultural Hukou, non‐agricultural Hukou, unified residence Hukou, do not have Hukou). Health‐related factors were systematically categorized into four domains: behavioral risk, subjective health assessments, physiological indicators, and chronic disease profiles. Behavioral risks included smoking status and alcohol consumption patterns. Smoking was recorded as a binary variable (yes/no), while alcohol intake was stratified into three categories: (l) I never had a drink, (2) I used to drink less than once a month, and (3) I used to drink more than once a month. Subjective health status was measured using two methodologies. Self‐health status was operationalized as a binary outcome, distinguishing between “excellent/very good/good” and “fair/poor” responses. Psychological well‐being was evaluated using the 10‐item Center for Epidemiologic Studies Depression Scale (CESD‐10), a validated tool scoring each item on a 0−3 Likert scale (total range: 0−30). Participants who scored ≥ 10 were classified as having clinically relevant depressive symptoms, consistent with the established cutoff thresholds. Sleep duration was derived from self‐reported nightly hours of sleep and categorized into tertiles: short (< 7 h), standard (> 7, ≤ 8 h), and extended (>8 h), reflecting evidence‐based sleep quality guidelines [22]. The chronic disease burden was assessed across 14 medically defined conditions, each recorded as a binary variable (yes/no). These included metabolic disorders (hypertension, dyslipidemia, diabetes), cardiovascular diseases (heart disease, stroke), neoplasms (cancer or malignant tumor), respiratory illnesses (chronic lung disease, asthma), gastrointestinal pathologies (stomach or other digestive disease), musculoskeletal conditions (arthritis or rheumatism), and neurocognitive impairments (emotional, nervous, or psychiatric problems, memory‐related disease), with diagnoses confirmed through medical history questionnaires [23].

### 2.3 Statistical Analysis

In this study, categorical variables will be presented as numerical percentages (%) and compared using chi‐square analysis (with Fisher's exact test if necessary). The mean ± standard deviation (SD) was used to describe normally distributed continuous variables. Non‐normally distributed continuous variables are expressed as median and interquartile range (IQR). Data analysis was performed using R version 4.2.0 and Empower Stats. Statistical significance was set at *p* < 0.05. Linear and multivariate logistic regression analyses were used, with SBP and DBP as continuous variables, to analyze the differences between continuous variables using linear regression. At the same time, based on the measurement values of SBP and DBP, blood pressure variables were used as categorical variables, and logistic regression was applied to analyze their association with dental visits. The characteristics of each group of participants were compared using diversified modeling methods to ensure the stability of the results.

To verify the potential association between dental visits and hypertension levels in middle‐aged and elderly people, we used the values of SBP and DBP as the basis, divided hypertension levels into normal, pre‐hypertension, and hypertension as exposure factors, and conducted multiple logistic regression analyses of three models. If *p* < 0.05 in covariate univariate analysis, it will be included in the multivariate model as a confounding factor. Model 1 did not undergo any variable adjustments. Model 2, we adjusted for age, gender, household registration, education, and marriage. In Model 3, we adjusted for age, gender, household registration, education, marriage, smoking, alcohol consumption, nighttime sleep duration, depression score, and 14 chronic diseases. Apply the GAM model to fit smooth curves, observe the linear or nonlinear relationship between SBP and DBP and dental visits after adjusting for all confounding factors, and use the log likelihood ratio test to detect linear or nonlinear correlation. Further testing of the robustness of the study through subgroup analysis and interaction analysis.

### 2.4 Ethics Approval and Consent to Participate

All data collection processes comply with the Biomedical Ethics Review Requirements of Peking University (IRB00001052‐11015). Each respondent signed an informed consent form before the survey, and their information was kept confidential.

## 3 Results

### 3.1 Demographic Characteristics by Hypertensive Status

After screening according to the inclusion and exclusion criteria, a total of 4770 participants were included in this study. Participants were divided into three groups based on their blood pressure status: normal, prehypertension, and hypertension. Preliminary analysis was conducted on their demographic characteristics using these groups, and detailed baseline features are shown in Table 1. The results showed that 1536 individuals had normal blood pressure levels, with an average age of 58.44 ± 8.43; 1666 patients with prehypertension, with an average age of 60.19 ± 8.70; 1568 hypertensive patients, with an average age of 62.81 ± 9.33. Among the 4770 middle‐aged and elderly people, the probability of early hypertension and hypertension patients going to dental clinic due to oral diseases was 28.66% and 27.69%, respectively. Compared with people with normal blood pressure, patients with hypertension are older, more male, married, with general/poor self‐health, and most have chronic diseases (hypertension, dyslipidemia, diabetes, etc.). Participants with normal blood pressure levels smoke and drink less alcohol than those with hypertension. The SBP/DBP of hypertensive patients was higher than that of non‐hypertensive patients (all *p* < 0.01).

**Table 1:** Baseline characteristics of groups by hypertensive status.

Variable	Normal (*n* = 1536)	pre‐hypertension (*n* = 1666)	Hypertension (*n* = 1568)	*p* value
Age (years)	58.44 ± 8.43	60.19 ± 8.70	62.81 ± 9.33	<0.001
Sex				<0.001
Man	673 (43.82%)	864 (51.86%)	786 (50.13%)	
Female	863 (56.19%)	802 (48.14%)	782 (49.87%)	
Hukou type				0.590
Agricultural Hukou	1233 (80.27%)	1320 (79.23%)	1212 (77.30%)	
Non‐agricultural Hukou	285 (18.56%)	324 (19.45%)	337 (21.49%)	
Unified Residence Hukou	17 (1.11%)	21 (1.26%)	18 (1.15%)	
Do not have Hukou	1 (0.07%)	1 (0.06%)	1 (0.06%)	
Educational level				0.208
Illiterate	647 (42.12%)	698 (41.90%)	681 (43.43%)	
Primary (include literate)	665 (43.29%)	766 (45.98%)	686 (43.75%)	
High school and above	224 (14.58%)	202 (12.13%)	201 (12.82%)	
Marital status				<0.001
Married with spouse present	1356 (88.28%)	1409 (84.57%)	1253 (79.91%)	
Married not living with spouse	56 (3.65%)	62 (3.72%)	61 (3.89%)	
Separated/divorced/widowed/never or Cohabitated	124 (8.07%)	195 (11.71%)	254 (16.20%)	
Self‐health status				0.044
Excellent/very good or good	352 (22.92%)	443 (26.59%)	376 (23.98%)	
Fair or poor	1184 (77.08%)	1223 (73.41%)	1192 (76.02%)	
CESD score				0.048
<10	1000 (65.10%)	1139 (68.37%)	1081 (68.94%)	
≥10	536 (34.90%)	527 (31.63%)	487 (31.06%)	
Hours of sleep at night				0.016
≤7	1189 (77.41%)	1246 (74.79%)	1138 (72.58%)	
>7, ≤8	254 (16.54%)	305 (18.31%)	295 (18.81%)	
>8	93 (6.06%)	115 (6.90%)	135 (8.61%)	
Smoking (By smoking, we mean smoking more than 100 cigarettes in your life?)				0.004
No	553 (36.00%)	677 (40.64%)	649 (41.39%)	
Yes	983 (64.00%)	989 (59.36%)	919 (58.61%)	
Drinking				<0.001
I never had a drink	366 (23.83%)	503 (30.19%)	428 (27.30%)	
I used to drink less than once a month	153 (9.96%)	121 (7.26%)	104 (6.63%)	
I used to drink more than once a month	1017 (66.21%)	1042 (62.55%)	1036 (66.07%)	
Hypertension				<0.001
Yes	129 (8.67%)	377 (23.16%)	629 (40.79%)	
No	1359 (91.33%)	1251 (76.84%)	913 (59.21%)	
Dyslipidemia				<0.001
Yes	99 (6.74%)	136 (8.51%)	192 (12.65%)	
No	1369 (93.26%)	1462 (91.49%)	1326 (87.35%)	
Diabetes or high blood sugar				0.019
Yes	65 (4.38%)	89 (5.55%)	103 (6.73%)	
No	1418 (95.62%)	1515 (94.45%)	1428 (93.27%)	
Cancer or malignant tumor				0.172
Yes	16 (1.08%)	8 (0.49%)	14 (0.92%)	
No	1467 (98.92%)	1614 (99.51%)	1516 (99.09%)	
Chronic lung diseases				0.256
Yes	131 (8.80%)	166 (10.22%)	161 (10.46%)	
No	1357 (91.20%)	1458 (89.78%)	1379 (89.55%)	
Liver disease				0.533
Yes	73 (4.93%)	79 (4.88%)	64 (4.17%)	
No	1409 (95.07%)	1539 (95.12%)	1472 (95.83%)	
Heart disease				<0.001
Yes	160 (10.77%)	166 (10.23%)	222 (14.44%)	
No	1325 (89.23%)	1456 (89.77%)	1315 (85.56%)	
Stroke				0.002
Yes	24 (1.61%)	26 (1.60%)	49 (3.19%)	
No	1464 (98.39%)	1598 (98.40%)	1487 (96.81%)	
Kidney disease				0.861
Yes	98 (6.58%)	101 (6.25%)	103 (6.72%)	
No	1392 (93.42%)	1515 (93.75%)	1430 (93.28%)	
Stomach or other digestive disease				<0.001
Yes	410 (27.43%)	384 (23.56%)	305 (19.79%)	
No	1085 (72.58%)	1246 (76.44%)	1236 (80.21%)	
Emotional, nervous, or psychiatric problems				0.230
Yes	25 (1.68%)	17 (1.05%)	17 (1.11%)	
No	1461 (98.32%)	1599 (98.95%)	1517 (98.89%)	
Memory‐related disease				0.093
Yes	11 (0.74%)	17 (1.05%)	24 (1.57%)	
No	1473 (99.26%)	1603 (98.95%)	1508 (98.43%)	
Arthritis or rheumatism				0.582
Yes	537 (35.563%)	554 (33.80%)	537 (34.76%)	
No	973 (64.44%)	1085 (66.20%)	1008 (65.24%)	
Asthma				0.354
Yes	44 (2.97%)	56 (3.46%)	60 (3.92%)	
No	1440 (97.04%)	1562 (96.54%)	1469 (96.08%)	
SBP (mmHg)	109.92 ± 7.36	128.74 ± 5.98	152.88 ± 18.92	<0.001
SBP categorical (mmHg)				<0.001
<120	1507 (98.11%)	43 (2.58%)	42 (2.68%)	
≥120, <140	29 (1.89%)	1623 (97.42%)	94 (6.00%)	
≥140	0 (0.00%)	0 (0.00%)	1432 (91.33%)	
DBP (mmHg)	65.95 ± 6.85	75.91 ± 7.31	88.28 ± 14.12	<0.001
DBP categorical (mmHg)				<0.001
<80	1527 (99.41%)	1113 (66.89%)	378 (24.11%)	
≥80, <90	9 (0.59%)	551 (33.11%)	551 (35.14%)	
≥90	0 (0.000%)	0 (0.000%)	639 (40.75%)	
In the past year, have you seen a dentist for dental care, including dentures?				0.006
No	310 (20.18%)	299 (17.95%)	247 (15.75%)	
Yes	1226 (79.82%)	1367 (82.05%)	1321 (84.25%)	

There were no significant differences between hypertensive patients and the normal blood pressure group in terms of household registration type, education level, cancer or tumor, chronic lung disease, liver chronic disease, kidney disease, mental illness, memory‐related disease, and asthma disease.

In addition, we conducted stratified demographic analysis using SBP (three groups: low SBP < 120 mmHg, medium: 120 ≤ SBP < 140 mmHg, high: SBP ≥ 140 mmHg) and DBP (three groups: low DBP < 80 mmHg, medium: 80 ≤ DBP < 90 mmHg, high: DBP ≥ 90 mmHg) to evaluate the impact of blood pressure measurements on the outcome variable (dental visits). Moreover, Table S1 and Table S2 present descriptions of the study population grouped by SBP and DBP, respectively.

### 3.2 Association Between Hypertension and Dental Visits

The association between dental visits and blood pressure shows parameter estimates of the association between hypertension, SBP, DBP, and dental visits, as shown in Table 2. After adjusting for all confounding variables, Model 3 showed that for every one SD increase in SBP, the likelihood of visiting a dentist increased by 6.65% (OR = 1.07, 95% CI = 0.98−1.16, *p* = 0.137). For every one SD increase in DBP, the likelihood of seeking dental treatment for oral diseases increases by 13.37% (OR = 1.13, 95% CI = 1.04−1.23, *p* = 0.004). Next, divide SBP and DBP into categorical variables to explore their association with dental visits. Although not statistically significant, both SBP and DBP show a positive increasing trend in their association with dental visits, meaning that as blood pressure indicators SBP and DBP increase, the likelihood of dental visits increases. Based on the SBP and DBP examination values, classify the SBP and DBP cut‐off points proposed in the JNC7 report. Throughout the entire adjustment process of Model 1, Model 2, and Model 3, hypertension was consistently associated with dental visits. In Model 3, the correlation strength between hypertension and oral diseases slightly weakened, but remained significant (OR = 1.35, 95% CI = 1.12−1.63, *p* = 0.001; OR = 1.36, 95% CI = 1.13−1.65, *p* = 0.001; OR = 1.30, 95% CI = 1.06−1.60, *p* = 0.012). It is worth noting that in all models, hypertension is positively correlated with dental visits, indicating that the association between blood pressure levels and the likelihood of dental visits is stable.

**Table 2:** Association between hypertensive status, as judged by SBP and DBP, and dental visits.

Exposure	Non‐adjusted	Adjust I	Adjust II
	OR (95% CI), *p* value	OR (95% CI), *p* value	OR (95% CI), *p* value
SBP (mmHg) Per 1 SD increase	**1.09 (1.01, 1.18) 0.020**	**1.09 (1.01, 1.18) 0.026**	1.07 (0.98, 1.16) 0.137
SBP categorical			
<120	1.00	1.00	1.00
≥120, <140	1.11 (0.93, 1.32) 0.251	1.09 (0.92, 1.30) 0.325	1.07 (0.88, 1.28) 0.504
≥140	**1.26 (1.05, 1.52) 0.015**	**1.26 (1.04, 1.53) 0.018**	1.20 (0.97, 1.48) 0.091
DBP (mmHg) Per 1 SD increase	**1.14 (1.05, 1.23) 0.001**	**1.14 (1.05, 1.23) 0.002**	**1.13 (1.04, 1.23) 0.004**
DBP categorical			
<80	1.00	1.00	1.00
≥80, <90	1.09 (0.91, 1.30) 0.370	1.08 (0.90, 1.29) 0.429	1.02 (0.84, 1.23) 0.855
≥90	1.20 (0.95, 1.51) 0.118	1.20 (0.95, 1.52) 0.122	1.17 (0.91, 1.49) 0.219
Hypertension status			
Normal	1.00	1.00	1.00
Pre‐hypertension	1.16 (0.97, 1.38) 0.108	1.14 (0.96, 1.37) 0.145	1.11 (0.92, 1.35) 0.2684
Hypertension	**1.35 (1.12, 1.63) 0.001**	**1.36 (1.13, 1.65) 0.001**	**1.30 (1.06, 1.60) 0.0121**

*Note:* Non‐adjusted model adjust for: None. Adjust I model adjust for: Sex; Age; Hukou Type; Educational level; Marital status. Adjust II model adjust for: Sex; Age; Hukou Type; Educational level; Marital status; Self‐health status; CESD Score; Hours of sleep at night; By smoking we mean smoking more than 100 cigarettes in your life?; Drinking; Hypertension; Dyslipidemia; Diabetes or high blood sugar; Cancer or malignant tumor; Chronic lung diseases; Liver disease; Heart disease; Stroke; Kidney disease; Stomach or other digestive disease; Emotional, nervous, or psychiatric problems; Memory‐related disease; Arthritis or rheumatism; Asthma.

### 3.3 Linear Association Between Hypertensive Status and Oral Health

**Figure 2:**
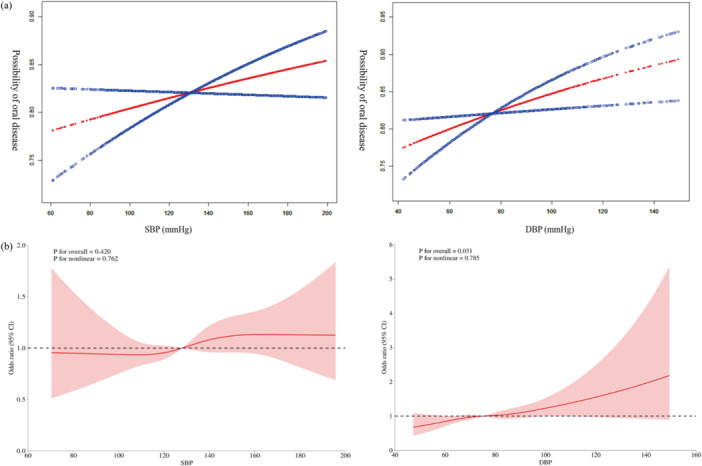
Linear association between hypertensive status and dental visits. (a) Curve‐fitting model; (b) Restricted cubic spline model.

**Figure 3:**
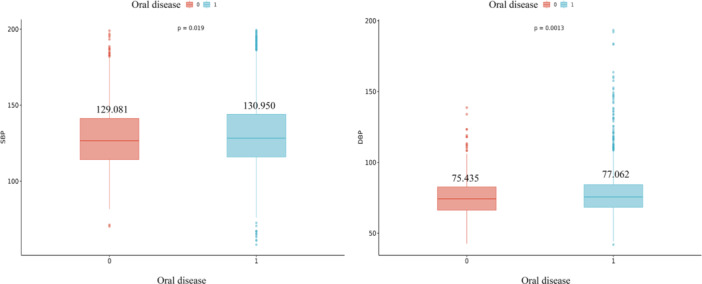
Box line plots showing the trend (median) and degree of dispersion of the SBP, DBP, and dental visits association data.

Smooth curve analysis was conducted based on the GAM to explore the potential linear/nonlinear relationship between blood pressure indicators (SBP and DBP) and dental visits. After adjusting for all covariates, the results showed a linear relationship between SBP and the likelihood of dental visits (Figure 2a). To ensure the stability of the linear results, we further used restricted cubic spline analysis (Figure 2b), which showed a trend consistent with the regression analysis. A box plot was used to illustrate the differences in the blood pressure monitoring data distribution between individuals with and without dental visits (Figure 3). Compared with participants without dental visits, participants with dental visits had higher levels of SBP (130.95 ± 21.27 mmHg vs. 129.08 ± 20.85 mmHg, *p* < 0.05) and DBP (77.06 ± 13.62 mmHg vs. 75.44 ± 12.54 mmHg, *p* < 0.05).

### 3.4 Subgroup Analyses

**Figure 4:**
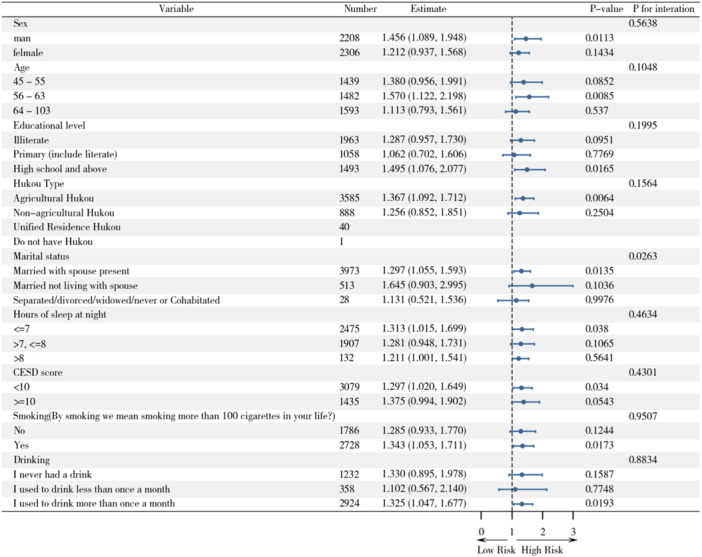
Subgroup analysis of the relationship between hypertensive status and dental visits.

In this study, the stability of the positive relationship between blood pressure levels and dental visits in different populations was investigated in subgroup analyses by sex, age, education, type of household, marital status, sleep duration, depression CESD scores, smoking status, and alcohol consumption (Figure 4). The results showed that after adjusting for covariates, the trend of OR values remained stable across strata, indicating a robust positive relationship between blood pressure level and dental visits, with no statistically significant interactions. Across populations, the association between basic social characterization factors and hypertension status showed a risk relationship (all OR values greater than 1). Specifically, gender analysis showed an increase in dental visits in hypertensive men (OR = 1.46, 95% CI: 1.09−1.94, *p* = 0.011). Across age groups, there was a significant increase of 57% in the 56−63 year age group (OR = 1.57, 95% CI = 1.12−2.20, *p* = 0.009), and marital status also influenced this association, with those who were married but did not live with a partner having an increased likelihood of dental visits (OR = 1.65, 95% CI = 0.90−3.00, *p* = 0.104). In addition, smokers had a 34.3% increase in dental visits compared to non‐smokers (OR = 1.34, 95% CI = 1.05−1.71, *p* = 0.017). Compared to non‐drinkers, those who drank alcohol more than once a week had a 32.5% increase in dental visits (OR = 1.33, 95% CI = 1.05−1.68, *p* = 0.019). However, these stratification factors did not show significant interactions (*p* > 0.05). These results suggest that age, sex, marital status, smoking, and drinking behaviors are important moderators of hypertension and dental visits, suggesting the need to focus on these factors when developing intervention strategies.

## 4 Discussion

Given that China's aging level has reached the upper middle level globally, this study focuses on exploring the relationship between hypertension and typical elderly chronic diseases, such as oral health in the process of population aging. In this study, we ultimately included 4770 participants for cross‐sectional analysis. The demographic analysis results show that most patients with hypertension are elderly married men, their self‐health status is general/poor, and they all suffer from chronic diseases (such as hypertension, dyslipidemia, and diabetes). Notably, compared to normal individuals, people with hypertension tend to smoke and drink more. Furthermore, we classified systolic and DBP into categorical variables and explored their relationship with dental visits. Logistic regression analysis showed a positive increasing trend in the correlation between systolic and DBP and dental visits. After adjusting for covariates in models 1, 2, and 3, hypertension showed a stable positive correlation with dental visits. The final curve fitting and restrictive cubic plot also showed a linear positive correlation between SBP, DBP, and dental visits. The above results suggest that individuals with high SBP and DBP should pay attention to their oral health issues. As blood pressure increases, their likelihood of seeking dental treatment also increases. In addition, relevant medical institutions should pay more attention to the blood pressure and oral health issues of middle‐aged and elderly people to prevent the mutual influence of chronic diseases and aggravate the course of the disease.

In recent years, the relationship between hypertension and oral health has received increasing attention, and various studies have explored the multifaceted interactions between these two chronic diseases. Langari et al. conducted a study on the elderly population to investigate how antihypertensive drugs affect oral health‐related quality of life. Their research findings indicate that the use of these drugs significantly affects the oral health attitudes of older adults, highlighting the need for healthcare providers to consider oral health when managing hypertension in this population [24]. Similarly, the characteristics of saliva in patients with hypertension showed a direct and significant relationship between hypertension and saliva viscosity but an inverse relationship with saliva pH. Hypertension can lower the pH value of hypertensive patients and increase saliva viscosity, resulting in changes in the quality and quantity of saliva secretion, and affecting the oral health and quality of life of patients [25]. Kim et al. also demonstrated that dental caries are associated with an increased risk of cardiovascular events in patients with hypertension; thus, oral health has a broad impact on overall health outcomes, particularly in the chronic patient population with hypertension [26]. A special population found that patients with elevated blood pressure had higher perceived stress and poorer oral health, revealing that mental health may also play a role in the oral health of hypertensive patients [27]. Moreover, the study found a significant association between elevated blood pressure and poor oral health (caries, missing and filled tooth [DMFT] index) [28]. Recent studies have also explored the role of periodontal disease in hypertension, and the results have shown that mild inflammation mediates the potential link between the two [29]. Then, it was emphasized that oral health status should be assessed as a potential risk factor for hypertension, and effective oral care should be used as an auxiliary lifestyle measure during hypertension management. Similarly, inflammatory markers have been identified as mediators of the relationship between periodontitis and uncontrolled hypertension, indicating that controlling inflammation may be crucial for addressing these two diseases [30]. The above literature indicates a correlation between hypertension and oral health, which is consistent with the results of this study, revealing that hypertension affects the risk of clinical oral diseases and social public health. Therefore, special attention should be paid to the oral health issues of hypertensive patients, especially in middle‐aged and elderly populations.

Relevant literature has studied the mechanism underlying the positive association between hypertension and oral health [31, 32]. On the one hand, periodontitis can increase blood pressure. Chronic periodontitis can cause pathogens to release endotoxins, activate systemic immune responses, and produce proinflammatory factors such as IL‐6 and TNF‐α. These inflammatory factors can damage endothelial function, promote vasoconstriction, and thus increase blood pressure [33]. In addition, studies have found that saliva and gingival crevicular fluid of periodontitis patients contain C‐reactive protein, which can reduce the bioavailability of nitric oxide (NO) after blood circulation, leading to a decrease in vasodilation ability and an increase in hypertension [34]. On the other hand, oral inflammation in hypertensive patients can also worsen. Scholars have found that the imbalance of oral microbiota in patients with hypertension leads to a decrease in NO producing bacteria, and the overgrowth of pathogenic bacteria exacerbates oral inflammation [35]. Compared with normotensive subjects, hypertensive individuals showed lower salivary concentrations and reduced presence of beneficial bacteria, such as *Neisseria flavescens* [36]. There is a bidirectional relationship between hypertension and oral diseases. This relationship is influenced not only by inflammation and oral microbiota but also by common risk factors such as smoking and alcohol consumption [37]. Therefore, it is essential to include blood pressure monitoring in the clinical management of patients with chronic diseases to provide a comprehensive evaluation of their oral health.

The advantages of this study are reflected in the following aspects. Firstly, this study used representative high‐quality survey data from China based on multi‐stage stratified sampling to ensure the universality and scientific of the analysis results. Secondly, the regression analysis results of this study indicate that for every one SD increase in hypertension, the likelihood of dental visits increases by 30% (OR = 1.30, 95% CI = 1.06−1.60, *p* = 0.012). Both curve fitting and restrictive cubic plots demonstrate a robust linear relationship between hypertension and dental visits. However, this study also has some shortcomings. First, the oral health assessment in this study is self‐reported and subjective, with individual differences that may lead to underestimation or overestimation of the association between oral health and hypertension. Second, observational design is prone to bias, and cross‐sectional studies cannot demonstrate association. More research is needed to confirm the relationship between hypertension and oral diseases in the middle‐aged and elderly population in China. And in this research method, participants with missing information were directly removed, which may affect sample representativeness and statistical efficacy, resulting in certain limitations. Meanwhile, the data collected in this study are limited, and some covariates such as dental care status, periodontal probing depth, and attachment loss were not included in the model. In the future, we will conduct further longitudinal studies, design more comprehensive data collection, control for a wider range of confounding factors, and delve deeper into the pathogenesis, thereby deepening our understanding of the relationship between hypertension and oral health.

## 5 Conclusions

The elderly are a high‐risk group for chronic diseases such as hypertension. Based on data from the CHARLS, this study examined the association between hypertension status diagnosed based on SBP and DBP and dental visits, revealing a robust linear positive correlation between hypertension and dental visits. Oral healthcare professionals need to increase the importance of oral treatment for elderly hypertensive patients and propose targeted and effective avoidance strategies.

## Author Contributions

M.A. and W.L. contributed equally to the study design, data analysis, and drafting of the manuscript, as well as the interpretation of the results. S.Y.L. participated in data collection and statistical analysis and reviewed and revised the manuscript. J.K.S., as the co‐corresponding author, provided expert guidance in clinical and epidemiological aspects and reviewed the structure and content of the manuscript. X.Y.G. and J.L.S. were responsible for the literature review and methodological support, providing critical feedback on the academic content of the paper. Z.C., as the corresponding author, oversaw the overall research, reviewed, and finalized the manuscript.

## Ethics Statement

All study methods involving human subjects complied with the Declaration of Helsinki guidelines. The foundational CHARLS study received approval from Peking University's Ethical Review Committee (IRB00001052‐1015), ensuring every participant from CHARLS gave their informed consent in writing.

## Consent

The authors have nothing to report.

## Conflicts of Interest

The authors declare no conflicts of interest.

## Supporting information


Supporting File 1

Supporting File 2

Supporting File 3

## Data Availability

The data that support the findings of this study are available in the China Health and Retirement Longitudinal Study (CHARLS) at https://charls.pku.edu.cn/. These data were derived from the following resources available in the public domain: —the China Health and Retirement Longitudinal Study (CHARLS), https://charls.pku.edu.cn/. The data sets generated during or analyzed during the current study are available in the CHARLS repository, https://charls.pku.edu.cn/.

## References

[1] C. Guo and X. Zheng , “Health Challenges and Opportunities for an Aging China,” American Journal of Public Health 108 (2018): 890–892, 10.2105/AJPH.2018.304444. 29874508 PMC5993367

[2] F. Liu , S. Song , X. Ye , et al., “Oral Health‐Related Multiple Outcomes of Holistic Health in Elderly Individuals: An Umbrella Review of Systematic Reviews and Meta‐Analyses,” Frontiers in Public Health 10 (2022): 1021104, 10.3389/fpubh.2022.1021104. 36388333 PMC9650948

[3] H. Wu , Y. Wang , H. Zhang , et al., “An Investigation Into the Health Status of the Elderly Population in China and the Obstacles to Achieving Healthy Aging,” Sci Rep‐Uk 14 (2024): 31123, 10.1038/s41598-024-82443-2. PMC11681201 39730900

[4] S. Kaptoge , L. Pennells , D. De Bacquer , et al., “World Health Organization Cardiovascular Disease Risk Charts: Revised Models to Estimate Risk in 21 Global Regions,” Lancet Global Health 7 (2019): e1332–e1345, 10.1016/S2214-109X(19)30318-3. 31488387 PMC7025029

[5] Z. Cai , Z. Gong , Z. Li , L. Li , and W. Kong , “Vascular Extracellular Matrix Remodeling and Hypertension,” Antioxid Redox Sign 34 (2021): 765–783, 10.1089/ars.2020.8110. 32460598

[6] S. S. Franklin , V. A. Lopez , N. D. Wong , et al., “Single Versus Combined Blood Pressure Components and Risk for Cardiovascular Disease: The Framingham Heart Study,” Circulation 119 (2009): 243–250, 10.1161/CIRCULATIONAHA.108.797936. 19118251 PMC3042701

[7] Z. Wang , Z. Chen , L. Zhang , et al., “Status of Hypertension in China: Results From the China Hypertension Survey,2012‐2015,” Circulation 137 (2018): 2344–2356, 10.1161/CIRCULATIONAHA.117.032380. 29449338

[8] Y. Zhan , J. Jiao , W. Jing , et al., “Association Between Periodontitis and Hypertension: Cross‐Sectional Survey from the Fourth National Oral Health Survey of China (2015‐2016),” BMJ Open 13 (2023): e068724, 10.1136/bmjopen-2022-068724. PMC10069577 36972967

[9] Q. H. Zhi , Y. Si , X. Wang , et al., “Determining the Factors Associated with Oral Health‐Related Quality of Life in Chinese Elders: Findings From the Fourth National Survey,” Community Dent Oral 50 (2022): 311–320, 10.1111/cdoe.12674. 34213027

[10] J. Jiao , W. Jing , Y. Si , et al., “The Prevalence and Severity of Periodontal Disease in Mainland China: Data from the Fourth National Oral Health Survey (2015‐2016),” Journal of Clinical Periodontology 48 (2021): 168–179, 10.1111/jcpe.13396. 33103285

[11] J. Gong , G. Wang , Y. Wang , et al., “Nowcasting and Forecasting the Care Needs of the Older Population in China: Analysis of Data From the China Health and Retirement Longitudinal Study (Charls),” Lancet Public Health 7 (2022): e1005–e1013, 10.1016/S2468-2667(22)00203-1. 36423656 PMC9741660

[12] K. Wu , W. M. Li , L. Q. Yan , S. C. Yang , Z. Yang , and C. J. Li , “[Study of the Relationship Between Oral Diseases and Depression Symptoms in Middle‐Aged and Older Adult Populations in China‐a Retrospective Study Based on Charls Data],” Sichuan Da Xue Xue Bao. Yi Xue Ban = Journal of Sichuan University. Medical Science Edition 52 (2021): 987–991, 10.12182/20211160106. 34841766 PMC10408816

[13] B. Y. Chen , W. Z. Lin , Y. L. Li , et al., “Characteristics and Correlations of the Oral and Gut Fungal Microbiome With Hypertension,” Microbiol Spectr 11 (2023): e0195622, 10.1128/spectrum.01956-22. 36475759 PMC9927468

[14] M. Chen , Y. Cai , J. Guo , et al., “Circ_0000284: A Risk Factor and Potential Biomarker for Prehypertension and Hypertension,” Hypertension Research 46 (2023): 720–729, 10.1038/s41440-022-01140-7. 36543889

[15] W. S. Santos , I. G. Solon , and L. Branco , “Impact of Periodontal Lipopolysaccharides on Systemic Health: Mechanisms, Clinical Implications, and Future Directions,” Mol Oral Microbiol 40 (2025): 117–127, 10.1111/omi.12490. 39604065

[16] M. Su , T. Zhang , W. Zhang , Z. Li , and X. Fan , “Decomposition Analysis on the Equity of Health Examination Utilization for the Middle‐Aged and Elderly People in China: Based on Longitudinal Charls Data from 2011 to 2018,” BMC Public Health 24 (2024): 998, 10.1186/s12889-024-18068-x. 38600464 PMC11312603

[17] Y. Zhao , Y. Hu , J. P. Smith , J. Strauss , and G. Yang , “Cohort Profile: The China Health and Retirement Longitudinal Study (Charls),” International Journal of Epidemiology 43 (2014): 61–68, 10.1093/ije/dys203. 23243115 PMC3937970

[18] J. Wei , X. Yin , Q. Liu , L. Tan , and C. Jia , “Association Between Hypertension and Cognitive Function: A Cross‐Sectional Study in People Over 45 Years Old in China,” Journal of Clinical Hypertension 20 (2018): 1575–1583, 10.1111/jch.13393. 30259624 PMC8031190

[19] S. Wang , R. Chen , Q. Liu , et al., “Comprehensive Treatment of Hypertension Middle‐Aged and Elderly People: Cross‐Sectional Survey Data From the China Health and Retirement Longitudinal Study (CHARLS),” Lancet 386 (2015): S67, 10.1016/S0140-6736(15)00648-0.

[20] A. V. Chobanian , G. L. Bakris , H. R. Black , et al., “The Seventh Report of the Joint National Committee on Prevention, Detection, Evaluation, and Treatment of High Blood Pressure: the JNC 7 report,” JAMA 289, no. 19 (2003): 2560–2572, 10.1001/jama.289.19.2560. 12748199

[21] Y. Ma , R. Hua , Z. Yang , B. Zhong , L. Yan , and W. Xie , “Different Hypertension Thresholds and Cognitive Decline: A Pooled Analysis of Three Ageing Cohorts,” BMC Medicine 19 (2021): 287, 10.1186/s12916-021-02165-4. 34724953 PMC8561998

[22] W. Li , A. Kondracki , P. Gautam , et al., “The Association Between Sleep Duration, Napping, and Stroke Stratified by Self‐Health Status Among Chinese People Over 65 Years Old from the China Health and Retirement Longitudinal Study,” Sleep & Breathing 25 (2021): 1239–1246, 10.1007/s11325-020-02214-x. 33067754

[23] Z. Y. Fan , Y. Yang , C. H. Zhang , R. Y. Yin , L. Tang , and F. Zhang , “Prevalence and Patterns of Comorbidity Among Middle‐Aged and Elderly People in China: A Cross‐Sectional Study Based on Charls Data,” International Journal of General Medicine 14 (2021): 1449–1455, 10.2147/IJGM.S309783. 33907449 PMC8071077

[24] S. F. Langari , S. R. Hosseini , A. Bijani , et al., “Association Between Antihypertensive Drugs and the Elderly's Oral Health‐ Related Quality of Life: Results of Amirkola Cohort Study,” Casp J Intern Med 13 (2022): 582–588, 10.22088/cjim.13.3.582. PMC9348220 35974945

[25] A. Mohiti , F. Eslami , and M. R. Dehestani , “Does Hypertension Affect Saliva Properties?,” Journal of Dentistry 21 (2020): 190–194, 10.30476/DENTJODS.2019.80992.0. 33062812 PMC7519935

[26] J. Kim , H. J. Kim , J. Jeon , and T. J. Song , “Association Between Oral Health and Cardiovascular Outcomes in Patients With Hypertension: A Nationwide Cohort Study,” Journal of Hypertension 40 (2022): 374–381, 10.1097/HJH.0000000000003022. 34670996

[27] S. Atif , U. Syed , M. Rafiq , A. Fatima , S. Rana , and M. Tariq , “Comparison of Perceived Stress and Oral Health Status Using Perceived Stress Scale and Dmft Index Between Healthy, Undiagnosed Hypertensive, and Known Hypertensive Dental Patients,” PLoS One 19 (2024): e0311645, 10.1371/journal.pone.0311645. 39388400 PMC11469613

[28] M. Samami , F. Joukar , S. Hassanipour , et al., “Hypertension and Dmft: Insights from the Persian Guilan Cohort Study,” BMC Oral Health 24 (2024): 1456, 10.1186/s12903-024-05236-z. 39616341 PMC11607915

[29] P. R. Del , L. Landi , G. Grassi , et al., “Hypertension and Periodontitis: A Joint Report by the Italian Society of Hypertension (Siia) and the Italian Society of Periodontology and Implantology (Sidp),” High Blood Press Car 28 (2021): 427–438, 10.1007/s40292-021-00466-6. PMC8484186 34562228

[30] Y. Chen , J. Zheng , D. Ni , D. Zhang , and H. Zhu , “The Correlation Between Periodontitis and Uncontrolled Hypertension is Mediated by Inflammatory Markers: Results From a Cross‐Sectional Study of Urban Elderly Population in Southeast China,” BMC Oral Health 23 (2023): 919, 10.1186/s12903-023-03680-x. 38001437 PMC10675948

[31] C. Darnaud , F. Thomas , B. Pannier , N. Danchin , and P. Bouchard , “Oral Health and Blood Pressure: The Ipc Cohort,” American Journal of Hypertension 28 (2015): 1257–1261, 10.1093/ajh/hpv025. 25780017

[32] P. Marito , Y. Hasegawa , K. Tamaki , et al., “The Association of Dietary Intake, Oral Health, and Blood Pressure in Older Adults: A Cross‐Sectional Observational Study,” Nutrients 14, no. 6 (2022): 1279–1297, 10.3390/nu14061279. 35334938 PMC8950359

[33] T. J. Guzik , R. Nosalski , P. Maffia , and G. R. Drummond , “Immune and Inflammatory Mechanisms in Hypertension,” Nature Reviews Cardiology 21 (2024): 396–416, 10.1038/s41569-023-00964-1. 38172242

[34] Y. Higashi , “Roles of Oxidative Stress and Inflammation in Vascular Endothelial Dysfunction‐Related Disease,” Antioxidants‐Basel 11 (2022): 1958–1973, 10.3390/antiox11101958. 36290681 PMC9598825

[35] P. Pignatelli , G. Fabietti , A. Ricci , A. Piattelli , and M. C. Curia , “How Periodontal Disease and Presence of Nitric Oxide Reducing Oral Bacteria Can Affect Blood Pressure,” International Journal of Molecular Sciences 21, no. 20 (2020): 7538–7552, 10.3390/ijms21207538. 33066082 PMC7589924

[36] P. Barbadoro , E. Ponzio , E. Coccia , et al., “Association Between Hypertension, Oral Microbiome and Salivary Nitric Oxide: A Case‐Control Study,” Nitric Oxide‐Biol Ch 106 (2021): 66–71, 10.1016/j.niox.2020.11.002. 33186726

[37] M. Czesnikiewicz‐Guzik , G. Osmenda , M. Siedlinski , et al., “Causal Association Between Periodontitis and Hypertension: Evidence from Mendelian Randomization and a Randomized Controlled Trial of Non‐Surgical Periodontal Therapy,” European Heart Journal 40 (2019): 3459–3470, 10.1093/eurheartj/ehz646. 31504461 PMC6837161

