# Supplementary material for: The Association of Hypertension Based on Systolic and Diastolic Blood Pressure (SBP and DBP) With Dental Visits in the Population Aged 45 and Older: Cross‐Section Study Results From the China Health and Retirement Longitudinal Study (CHARLS)
Source: Clin Cardiol. 2026 Jul 9;49(7):e70406. doi: 10.1002/clc.70406 (PMC13349036; doi:10.1002/clc.70406)
Supplement: Supplementary file 2 — Supporting File 2 [file CLC-49-e70406-s003.docx]

**Table S1** Description of the study population grouped by SBP

| SBP categorical | <120 | >=120, <140 | >=140 | P-value |
| --- | --- | --- | --- | --- |
| N | 1592 | 1746 | 1432 |  |
| Age | 58.405 ± 8.384 | 60.092 ± 8.709 | 63.286 ± 9.307 | <0.001 |
| Sex |  |  |  | <0.001 |
| man | 699 (43.907%) | 903 (51.718%) | 721 (50.349%) |  |
| felmale | 893 (56.093%) | 843 (48.282%) | 711 (49.651%) |  |
| Educational level |  |  |  | 0.271 |
| Illiterate | 667 (41.897%) | 726 (41.581%) | 633 (44.204%) |  |
| Primary (include literate) | 697 (43.781%) | 798 (45.704%) | 622 (43.436%) |  |
| High school and above | 228 (14.322%) | 222 (12.715%) | 177 (12.360%) |  |
| Marital status |  |  |  | <0.001 |
| Married with spouse present | 1398 (87.814%) | 1485 (85.052%) | 1135 (79.260%) |  |
| Married not living with spouse | 59 (3.706%) | 66 (3.780%) | 54 (3.771%) |  |
| Separated/divorced/widowed/never or Cohabitated | 135 (8.480%) | 195 (11.168%) | 243 (16.969%) |  |
| Self-health status |  |  |  | 0.061 |
| Excellent /Very good or Good | 360 (22.613%) | 456 (26.117%) | 355 (24.791%) |  |
| Fair or Poor | 1232 (77.387%) | 1290 (73.883%) | 1077 (75.209%) |  |
| CESD score |  |  |  | 0.04 |
| <10 | 1036 (65.075%) | 1198 (68.614%) | 986 (68.855%) |  |
| >=10 | 556 (34.925%) | 548 (31.386%) | 446 (31.145%) |  |
| Hours of sleep at night |  |  |  | 0.007 |
| <=7 | 1227 (77.073%) | 1318 (75.487%) | 1028 (71.788%) |  |
| >7, <=8 | 266 (16.709%) | 311 (17.812%) | 277 (19.344%) |  |
| >8 | 99 (6.219%) | 117 (6.701%) | 127 (8.869%) |  |
| Smoking(By smoking we mean smoking more than 100 cigarettes in your life?) |  |  |  | 0.004 |
| No | 575 (36.118%) | 709 (40.607%) | 595 (41.550%) |  |
| Yes | 1017 (63.882%) | 1037 (59.393%) | 837 (58.450%) |  |
| Drinking |  |  |  | <0.001 |
| I never had a drink | 382 (23.995%) | 523 (29.954%) | 392 (27.374%) |  |
| I used to drink less than once a month | 158 (9.925%) | 129 (7.388%) | 91 (6.355%) |  |
| I used to drink more than once a month | 1052 (66.080%) | 1094 (62.658%) | 949 (66.271%) |  |
| Hypertension |  |  |  | <0.001 |
| Yes | 140 (9.061%) | 401 (23.533%) | 594 (42.158%) |  |
| No | 1405 (90.939%) | 1303 (76.467%) | 815 (57.842%) |  |
| Dyslipidemia |  |  |  | <0.001 |
| Yes | 99 (6.492%) | 150 (8.971%) | 178 (12.833%) |  |
| No | 1426 (93.508%) | 1522 (91.029%) | 1209 (87.167%) |  |
| Diabetes or high blood sugar |  |  |  | 0.006 |
| Yes | 66 (4.286%) | 93 (5.546%) | 98 (6.995%) |  |
| No | 1474 (95.714%) | 1584 (94.454%) | 1303 (93.005%) |  |
| Cancer or malignant tumor |  |  |  | 0.465 |
| Yes | 16 (1.038%) | 11 (0.649%) | 11 (0.786%) |  |
| No | 1525 (98.962%) | 1683 (99.351%) | 1389 (99.214%) |  |
| Chronic lung diseases |  |  |  | 0.384 |
| Yes | 139 (8.997%) | 176 (10.365%) | 143 (10.149%) |  |
| No | 1406 (91.003%) | 1522 (89.635%) | 1266 (89.851%) |  |
| Liver disease |  |  |  | 0.276 |
| Yes | 75 (4.873%) | 86 (5.080%) | 55 (3.917%) |  |
| No | 1464 (95.127%) | 1607 (94.920%) | 1349 (96.083%) |  |
| Heart disease |  |  |  | 0.009 |
| Yes | 168 (10.888%) | 183 (10.796%) | 197 (14.011%) |  |
| No | 1375 (89.112%) | 1512 (89.204%) | 1209 (85.989%) |  |
| Stroke |  |  |  | 0.001 |
| Yes | 22 (1.423%) | 31 (1.826%) | 46 (3.276%) |  |
| No | 1524 (98.577%) | 1667 (98.174%) | 1358 (96.724%) |  |
| Kidney disease |  |  |  | 0.856 |
| Yes | 101 (6.529%) | 106 (6.276%) | 95 (6.771%) |  |
| No | 1446 (93.471%) | 1583 (93.724%) | 1308 (93.229%) |  |
| Stomach or other digestive disease |  |  |  | <0.001 |
| Yes | 427 (27.513%) | 399 (23.388%) | 273 (19.389%) |  |
| No | 1125 (72.487%) | 1307 (76.612%) | 1135 (80.611%) |  |
| Emotional, nervous, or psychiatric problems |  |  |  | 0.481 |
| Yes | 24 (1.554%) | 19 (1.125%) | 16 (1.140%) |  |
| No | 1520 (98.446%) | 1670 (98.875%) | 1387 (98.860%) |  |
| Memory-related disease |  |  |  | 0.089 |
| Yes | 11 (0.714%) | 19 (1.122%) | 22 (1.569%) |  |
| No | 1530 (99.286%) | 1674 (98.878%) | 1380 (98.431%) |  |
| Arthritis or rheumatism |  |  |  | 0.789 |
| Yes | 554 (35.354%) | 588 (34.286%) | 486 (34.419%) |  |
| No | 1013 (64.646%) | 1127 (65.714%) | 926 (65.581%) |  |
| Asthma |  |  |  | 0.288 |
| Yes | 47 (3.050%) | 56 (3.310%) | 57 (4.077%) |  |
| No | 1494 (96.950%) | 1636 (96.690%) | 1341 (95.923%) |  |
| Hukou Type |  |  |  | 0.763 |
| Agricultural Hukou | 1272 (79.899%) | 1384 (79.267%) | 1109 (77.444%) |  |
| Non-agricultural Hukou | 301 (18.907%) | 339 (19.416%) | 306 (21.369%) |  |
| Unified Residence Hukou | 18 (1.131%) | 22 (1.260%) | 16 (1.117%) |  |
| Do not have Hukou | 1 (0.063%) | 1 (0.057%) | 1 (0.070%) |  |
| Oral disease (In the past year, have you seen a dentist for dental care, including dentures?) |  |  |  | 0.051 |
| l disease | 311 (19.535%) | 314 (17.984%) | 231 (16.131%) |  |
| Visiting with an oral disease | 1281 (80.465%) | 1432 (82.016%) | 1201 (83.869%) |  |
