# Supplementary material for: The Association of Hypertension Based on Systolic and Diastolic Blood Pressure (SBP and DBP) With Dental Visits in the Population Aged 45 and Older: Cross‐Section Study Results From the China Health and Retirement Longitudinal Study (CHARLS)
Source: Clin Cardiol. 2026 Jul 9;49(7):e70406. doi: 10.1002/clc.70406 (PMC13349036; doi:10.1002/clc.70406)
Supplement: Supplementary file 3 — Supporting File 3 [file CLC-49-e70406-s001.docx]

**Table S2** Description of the study population grouped by DBP

| DBP categorical | <80 | >=80, <90 | >=90 | P-value |
| --- | --- | --- | --- | --- |
| N | 3018 | 1111 | 639 |  |
| Age | 60.980 ± 9.270 | 60.261 ± 8.501 | 58.588 ± 8.282 | <0.001 |
| Sex |  |  |  | <0.001 |
| man | 1406 (46.587%) | 579 (52.115%) | 337 (52.739%) |  |
| felmale | 1612 (53.413%) | 532 (47.885%) | 302 (47.261%) |  |
| Educational level |  |  |  | <0.001 |
| Illiterate | 1347 (44.632%) | 445 (40.054%) | 234 (36.620%) |  |
| Primary (include literate) | 1291 (42.777%) | 522 (46.985%) | 303 (47.418%) |  |
| High school and above | 380 (12.591%) | 144 (12.961%) | 102 (15.962%) |  |
| Marital status |  |  |  | 0.206 |
| Married with spouse present | 2555 (84.659%) | 942 (84.788%) | 519 (81.221%) |  |
| Married not living with spouse | 105 (3.479%) | 44 (3.960%) | 30 (4.695%) |  |
| Separated/divorced/widowed/never or Cohabitated | 358 (11.862%) | 125 (11.251%) | 90 (14.085%) |  |
| Self-health status |  |  |  | 0.213 |
| Excellent /Very good or Good | 753 (24.950%) | 278 (25.023%) | 139 (21.753%) |  |
| Fair or Poor | 2265 (75.050%) | 833 (74.977%) | 500 (78.247%) |  |
| CESD score |  |  |  | 0.527 |
| <10 | 2020 (66.932%) | 760 (68.407%) | 439 (68.701%) |  |
| >=10 | 998 (33.068%) | 351 (31.593%) | 200 (31.299%) |  |
| Hours of sleep at night |  |  |  | 0.003 |
| <=7 | 2298 (76.143%) | 813 (73.177%) | 460 (71.987%) |  |
| >7, <=8 | 496 (16.435%) | 232 (20.882%) | 126 (19.718%) |  |
| >8 | 224 (7.422%) | 66 (5.941%) | 53 (8.294%) |  |
| Smoking(By smoking we mean smoking more than 100 cigarettes in your life?) |  |  |  | 0.011 |
| No | 1143 (37.873%) | 456 (41.044%) | 279 (43.662%) |  |
| Yes | 1875 (62.127%) | 655 (58.956%) | 360 (56.338%) |  |
| Drinking |  |  |  | <0.001 |
| I never had a drink | 750 (24.851%) | 346 (31.143%) | 200 (31.299%) |  |
| I used to drink less than once a month | 242 (8.019%) | 85 (7.651%) | 51 (7.981%) |  |
| I used to drink more than once a month | 2026 (67.131%) | 680 (61.206%) | 388 (60.720%) |  |
| Hypertension |  |  |  | <0.001 |
| Yes | 522 (17.791%) | 352 (32.176%) | 260 (41.401%) |  |
| No | 2412 (82.209%) | 742 (67.824%) | 368 (58.599%) |  |
| Dyslipidemia |  |  |  | <0.001 |
| Yes | 231 (7.993%) | 127 (11.803%) | 69 (11.201%) |  |
| No | 2659 (92.007%) | 949 (88.197%) | 547 (88.799%) |  |
| Diabetes or high blood sugar |  |  |  | 0.277 |
| Yes | 157 (5.384%) | 70 (6.487%) | 30 (4.831%) |  |
| No | 2759 (94.616%) | 1009 (93.513%) | 591 (95.169%) |  |
| Cancer or malignant tumor |  |  |  | 0.527 |
| Yes | 26 (0.889%) | 6 (0.553%) | 6 (0.965%) |  |
| No | 2900 (99.111%) | 1079 (99.447%) | 616 (99.035%) |  |
| Chronic lung diseases |  |  |  | 0.869 |
| Yes | 285 (9.710%) | 108 (9.908%) | 65 (10.400%) |  |
| No | 2650 (90.290%) | 982 (90.092%) | 560 (89.600%) |  |
| Liver disease |  |  |  | 0.824 |
| Yes | 138 (4.720%) | 47 (4.340%) | 31 (4.944%) |  |
| No | 2786 (95.280%) | 1036 (95.660%) | 596 (95.056%) |  |
| Heart disease |  |  |  | 0.13 |
| Yes | 329 (11.225%) | 131 (12.074%) | 88 (14.058%) |  |
| No | 2602 (88.775%) | 954 (87.926%) | 538 (85.942%) |  |
| Stroke |  |  |  | 0.108 |
| Yes | 53 (1.805%) | 31 (2.857%) | 15 (2.400%) |  |
| No | 2883 (98.195%) | 1054 (97.143%) | 610 (97.600%) |  |
| Kidney disease |  |  |  | 0.184 |
| Yes | 176 (6.007%) | 79 (7.295%) | 47 (7.532%) |  |
| No | 2754 (93.993%) | 1004 (92.705%) | 577 (92.468%) |  |
| Stomach or other digestive disease |  |  |  | 0.059 |
| Yes | 727 (24.669%) | 235 (21.619%) | 136 (21.587%) |  |
| No | 2220 (75.331%) | 852 (78.381%) | 494 (78.413%) |  |
| Emotional, nervous, or psychiatric problems |  |  |  | 0.787 |
| Yes | 35 (1.198%) | 16 (1.473%) | 8 (1.278%) |  |
| No | 2887 (98.802%) | 1070 (98.527%) | 618 (98.722%) |  |
| Memory-related disease |  |  |  | 0.922 |
| Yes | 32 (1.094%) | 12 (1.107%) | 8 (1.280%) |  |
| No | 2893 (98.906%) | 1072 (98.893%) | 617 (98.720%) |  |
| Arthritis or rheumatism |  |  |  | 0.216 |
| Yes | 1057 (35.625%) | 363 (33.090%) | 208 (33.121%) |  |
| No | 1910 (64.375%) | 734 (66.910%) | 420 (66.879%) |  |
| Asthma |  |  |  | 0.879 |
| Yes | 104 (3.557%) | 36 (3.330%) | 20 (3.205%) |  |
| No | 2820 (96.443%) | 1045 (96.670%) | 604 (96.795%) |  |
| Hukou Type |  |  |  | 0.485 |
| Agricultural Hukou | 2388 (79.125%) | 881 (79.298%) | 494 (77.308%) |  |
| Non-agricultural Hukou | 595 (19.715%) | 211 (18.992%) | 140 (21.909%) |  |
| Unified Residence Hukou | 33 (1.093%) | 18 (1.620%) | 5 (0.782%) |  |
| Do not have Hukou | 2 (0.066%) | 1 (0.090%) | 0 (0.000%) |  |
| In the past year, have you seen a dentist for dental care, including dentures? |  |  |  | 0.246 |
| No | 561 (18.588%) | 193 (17.372%) | 102 (15.962%) |  |
| Yes | 2457 (81.412%) | 918 (82.628%) | 537 (84.038%) |  |
